# Spatial separation of oxidation and reduction co-catalysts for efficient charge separation: Pt@TiO_2_@MnO_*x*_ hollow spheres for photocatalytic reactions†
†Electronic supplementary information (ESI) available. See DOI: 10.1039/c5sc04163e


**DOI:** 10.1039/c5sc04163e

**Published:** 2015-11-26

**Authors:** Ang Li, Tuo Wang, Xiaoxia Chang, Weiting Cai, Peng Zhang, Jijie Zhang, Jinlong Gong

**Affiliations:** a Key Laboratory for Green Chemical Technology of Ministry of Education , School of Chemical Engineering and Technology , Collaborative Innovation Center of Chemical Science and Engineering , Tianjin University , Weijin Road 92 , Tianjin , 300072 , P. R. China . Email: jlgong@tju.edu.cn

## Abstract

Efficient charge separation is a critical factor for solar energy conversion by heterogeneous photocatalysts. This paper describes the complete spatial separation of oxidation and reduction cocatalysts to enhance the efficacy of charge separation and surface reaction. Specifically, we design Pt@TiO_2_@MnO_*x*_ hollow spheres (PTM-HSs) with Pt and MnO_*x*_ loaded onto the inner and outer surface of TiO_2_ shells, respectively. Pt favours electron trapping, while MnO_*x*_ tends to collect holes. Upon generation from TiO_2_, electrons and holes flow inward and outward of the spherical photocatalyst, accumulating on the corresponding cocatalysts, and then take part in redox reactions. Combined with other advantages, such as the large surface area and appropriate pore size, the PTM-HSs exhibit high efficiency for the photocatalytic oxidation of water and benzyl alcohol. The mechanism of the oxidation process of benzyl alcohol over the photocatalyst is also presented.

## Abstract 2

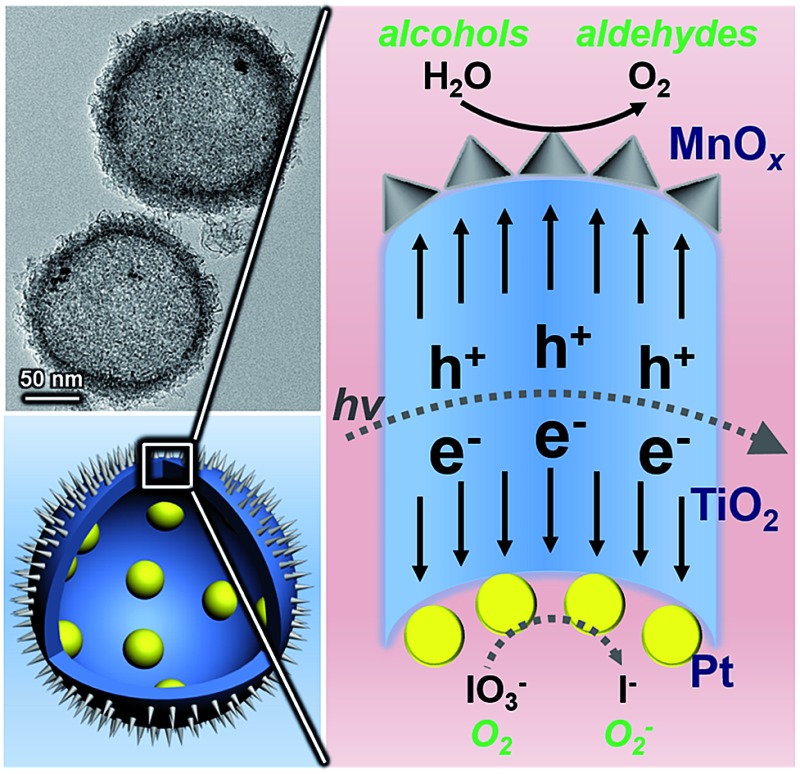
The design and synthesis of fine Pt@TiO_2_@MnO_*x*_ hollow spheres is described. Pt and MnO_*x*_ are spatially separated by TiO_2_ shells. The catalyst exhibits high efficiency of charge-separation and surface-reaction.

## Introduction

Efficient charge separation is a critical factor for solar energy conversion by semiconductor-based systems. It is crucial for increasing the performance of solar cells, photoelectrochemical and photocatalytic systems.^1,2^ As an important application, photocatalytic oxidation requires exceptionally efficient charge separation. Within photooxidation reactions, the photooxidation of water into O_2_ has long been considered as the bottleneck of the water-splitting process, so the invention of highly active water oxidation catalysts is a key step in the development of light-driven water splitting.^3^ In addition, the photocatalytic oxidation system can also be used to produce highly valued chemicals such as aldehydes and ketones. Compared with traditional methods with high temperature, toxic oxidants and hazardous waste,^4^ photocatalytic oxidation processes can be performed under mild and environmentally friendly conditions. Substances unstable at high temperatures may also be synthesized by such selective light-assisted processes.^4^ Recently, the emergence of a new family of metal-free polymer photocatalysts provided fine methods to enhance charge separation.^5,6^ For instance, heterojunctions between carbon nitride and sulfur-mediated carbon nitride have been constructed to promote photocatalytic reactions.^6^ Besides, many other strategies were adopted to improve charge separation, while the results remain unsatisfactory because of diverse problems.^1,7–9^

Cocatalyst loading has been proved to be an effective approach to accelerate charge separation.^1^ Cocatalysts can be designed for oxidation and reduction purposes. Oxidation cocatalysts tend to capture holes (PbO_2_, MnO_*x*_ and PdS,^10^ *etc.*), while reduction cocatalysts prefer electrons (Pt, Pd,^1^ *etc.*). Meanwhile, they can promote the surface reaction of the redox process.^11^ Yu *et al.* have reported the fabrication of TiO_2_ nanosheets loaded with Pt. Compared with pure TiO_2_ nanosheets, the photocatalytic activity of Pt decorated nanosheets was obviously improved.^2^

Simultaneous loading of oxidation and reduction cocatalysts could further improve the photocatalytic activity. Colón *et al.* fabricated Pt–TiO_2_/g-C_3_N_4_–MnO_*x*_ composites and successfully proved that Pt and MnO_*x*_ trap electrons and holes, respectively, leading to a further separation of charges.^12^ However, both reduction and oxidation cocatalysts are randomly distributed in this case, which results in a random flow direction of charge carriers when they migrate to the cocatalysts. In such a scenario, the possibility of electron–hole recombination will be increased. In addition, the short distance between the reduction and oxidation cocatalysts is unfavorable for redox reactions, for close redox sites may lead to severe back-reactions.

Spatial separation of oxidation and reduction cocatalysts is a method to solve this problem, yet limited success has been reported. Carbon nitride nanosheets have been fabricated to separate different cocatalysts, promoting the locally-incompatible oxidation and reduction reactions on the two surfaces.^5^ In addition, Pt particles (reduction cocatalyst) and MnO_*x*_ particles (oxidation cocatalyst) have been nicely deposited on different facets of BiVO_4_, exhibiting increased performance when compared to the randomly deposited counterparts.^1^ To achieve more complete spatial separation of oxidation and reduction cocatalysts, as well as to make full use of the photocatalyst, we design and synthesize Pt@TiO_2_@MnO_*x*_ hollow spheres (PTM-HSs) with Pt particles and MnO_*x*_ loaded onto the inner and outer surfaces of TiO_2_ hollow spheres, respectively. Different cocatalysts can be completely separated by the TiO_2_ shells without intermixing. The photocatalytic material can be fully used owing to the thin shell (about 40–60 nm) of TiO_2_ hollow spheres. TiO_2_ is chosen in this study since it serves as an excellent benchmark photocatalyst for comparison purposes.^13–15^ Additionally, as a model structure, this concept (*i.e.*, different cocatalysts are separated by TiO_2_ shells completely) can be extended to other redox cocatalysts such as Au, Ag, PbO_2_, *etc.* ^1^

**Fig. 1:**
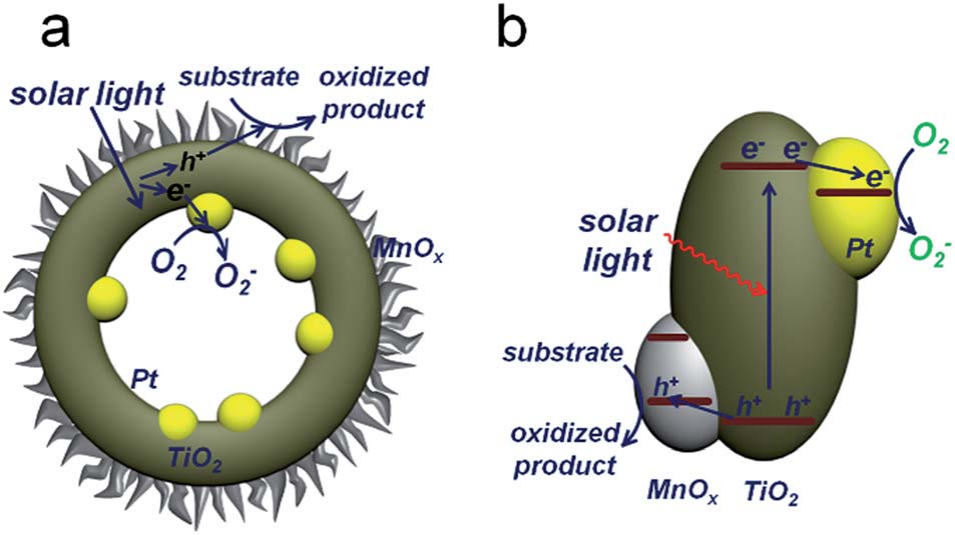
Proposed mechanism for photocatalytic oxidation by PTM-HSs. Pt and MnO_*x*_ are spatially separated by TiO_2_. (a) The reaction process. (b) Band structure of the catalyst.

Upon generation from TiO_2_, electrons and holes will flow inward and outward of the spherical TiO_2_ shells, respectively, accumulating on corresponding cocatalysts and then taking part in redox reactions (Fig. 1). Electrons can promote the photo-reduction, while holes participate in the photo-oxidation.^16^ Thus, the oxidation of benzyl alcohol accompanied by the elimination of electrons by O_2_ is described in Fig. 1a. The reduction and oxidation reactions take place at different sides of the TiO_2_ shells, which is critical for effective charge separation. In addition, light will pass through the thin TiO_2_ shell and keep reflecting in the cavity, increasing the scattering length for enhanced light-absorption. Combined with the large surface area, the PTM-HSs exhibit high efficiency for the photocatalytic oxidation of benzyl alcohol, a representative alcohol containing phenyl groups. Generally, the appropriate catalysts for benzyl alcohol oxidation can also perform well in the oxidation of other similar chemicals.^17^ Thus, the current photoredox catalysis system can be expanded for other relevant organic synthesis of fine chemicals. Additionally, we propose a two-step photooxidation mechanism for PTM-HSs, which may provide inspiration for the design of similar catalysts with high activity.

## Results and discussion

**Fig. 2:**
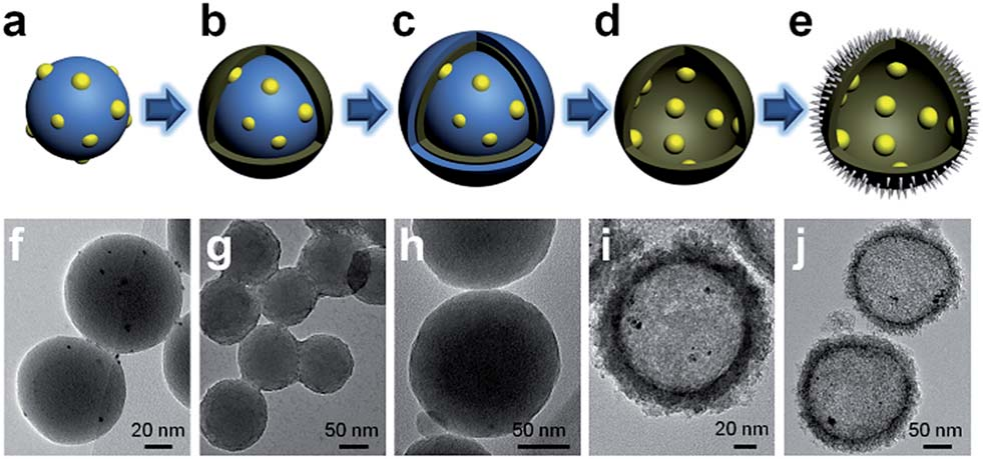
Schematic illustration and TEM images of the formation process of PTM-HSs. (a) and (f) Pt loaded onto SiO_2_ nanospheres to form SiO_2_–Pt. (b) and (g) TiO_2_ layers were coated on SiO_2_–Pt to form SiO_2_–Pt@TiO_2_. (c) and (h) SiO_2_ protective layers were coated on SiO_2_–Pt@TiO_2_ to form SiO_2_–Pt@TiO_2_@SiO_2_. (d) and (i) PT-HSs. (e) and (j) PTM-HSs. The outermost spine-like layer in image (j) is the MnO_*x*_ layer.

The synthesis of PTM-HSs (Fig. 2) starts from SiO_2_ nanospheres prepared by a modified Stöber method, with H_2_PtCl_6_ adsorbed on the surface of SiO_2_.^18^ Then they were calcined at 500 °C for 2 hours (h) under H_2_ atmosphere to form Pt particles anchored on SiO_2_ (Fig. 2a and f). Subsequent coating of an amorphous TiO_2_ shell was performed by the hydrolysis of titanium *tert*-butoxide (TBOT) (Fig. 2b and g). Then the samples were calcined to improve the crystallinity of TiO_2_. It should be noted that direct calcination would destroy the structure of the TiO_2_ shells (Fig. S2†). To preserve the morphology, another layer of SiO_2_ was added as a coating to form the outermost protective layer (Fig. 2c and h). After calcination, the outer and inner silica layers were removed by NaOH (1.67 M) etching at 70 °C for 8 h to form Pt@TiO_2_ hollow spheres (PT-HSs) (Fig. 2d and i). Finally, MnO_*x*_ was selectively deposited on the outer surface of PT-HSs to form PTM-HSs (Fig. 2e and j and S3†) by a photo-deposition method (Fig. S1†).

**Fig. 3:**
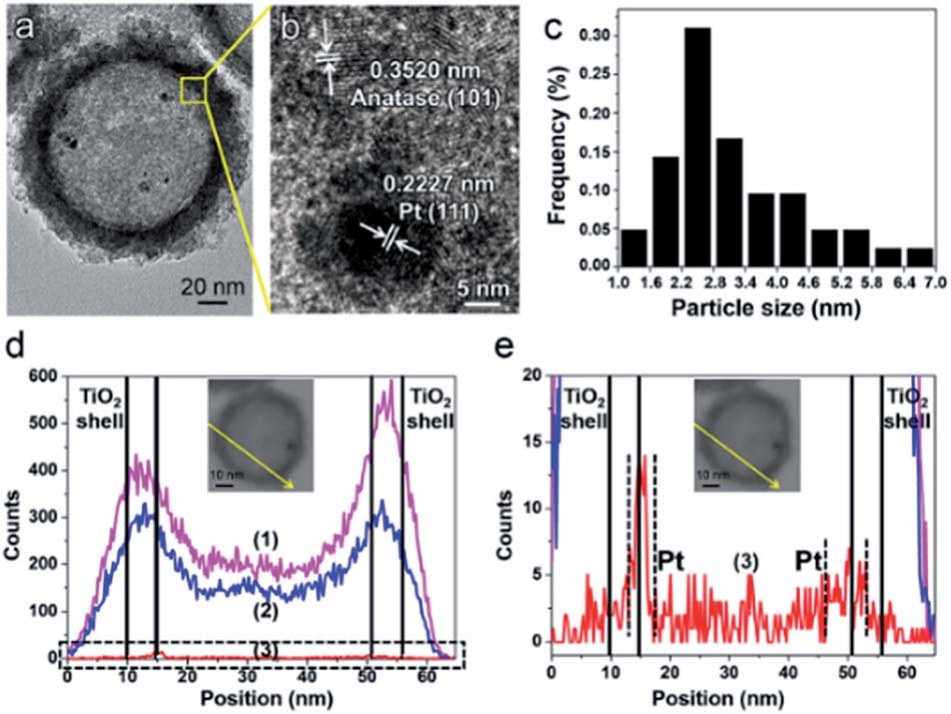
The confirmation of PT-HS structures. (a) TEM image of a PT-HS. (b) HRTEM image of the area near the inner surface of the TiO_2_ shell. (c) The distribution of the particle size. (d) EDS line scan of a PT-HS. The part in the dotted pane is magnified in image (e). (e) The magnified patterns of the EDS line scan. Inset in image (d) and (e): the path of the EDS line scan. Curves (1), (2) and (3) in image (d) and (e) refer to the signals of Ti, O and Pt, respectively.

The successful synthesis of the PT-HSs is further validated. Fig. 3a shows that most Pt particles with an average size of *ca.* 3.2 ± 1.3 nm (Fig. 3c) are uniformly loaded onto the inner surface of TiO_2_ shells. A few Pt particles agglomerate to form larger ones. The high resolution transmission electron microscopy (HRTEM) image of the area near the inner surface of TiO_2_ shells (Fig. 3b) shows that the lattice spacing of 0.2227 nm and 0.3520 nm match well with the (111) planes of Pt and the (101) planes of anatase. It can be seen that a hexagonal Pt particle is coated by a layer of TiO_2_ (Fig. 3b), resulting from the strong interaction between Pt and TiO_2_. A STEM-energy-dispersive X-ray spectroscopy (EDS) line scan was performed through the center of an individual PT-HS particle (inset in Fig. 3d and e), which further confirms the position of Pt particles relative to the TiO_2_ shell. Fig. 3d shows strong signals of Ti and O at the edge of the PT-HS, which reveals a typical structure of TiO_2_ hollow spheres. The TiO_2_ shell is marked in Fig. 3d by two groups of vertical lines, indicating the wall of the TiO_2_ shell based on Ti and O signals in the EDS line scan. Fig. 3e shows the magnified Pt EDS signal, demonstrating that Pt particles are loaded onto the inner surface of the TiO_2_ shell. It is not possible to use an EDS area scan to perform such confirmation because of the low loading of Pt (1%, measured by ICP) (Fig. S4d†).

**Fig. 4:**
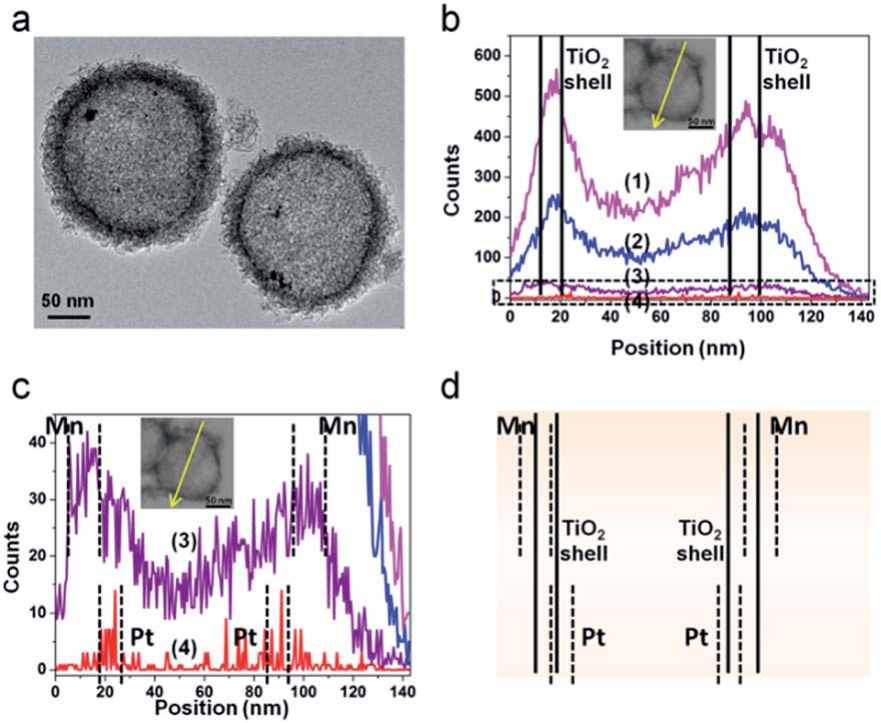
The confirmation of PTM-HS structures. (a) TEM image of PTM-HSs. (b) EDS line scan of a PTM-HS. The part in the dotted pane is magnified in image c. (c) The magnified patterns of the EDS line scan. (d) The relative positions of Mn, Pt and the TiO_2_ shell. Inset in (b) and (c): the path of the EDS line scan. Curves (1), (2), (3) and (4) in images (b) and (c) refer to the signals of Ti, O, Mn and Pt, respectively.

The structure of the PTM-HSs is confirmed by the same method (Fig. 4), where MnO_*x*_ is identified on the outer surface of TiO_2_ shells. The universal existence of PTM-HSs can be demonstrated by the TEM image of a larger area (Fig. S3†).

The pore size and Brunauer–Emmett–Teller (BET) surface area of PTM-HSs are measured by N_2_ adsorption, and determined to be 5.0 nm (average pore size) and 298 m^2^ g^–1^, respectively (Fig. S5a†). The results indicate a mesoporous structure, which is the prerequisite for effective immigration of reactants. Additionally, X-ray diffraction (XRD, Fig. S5b†) and HRTEM of the spine-like layer (Fig. S5c and d†) prove that the PTM-HSs consist of Pt, anatase and MnO_*x*_, where *x* is between 1.0 and 2.0. Highly crystallized anatase instead of a rutile shell is formed, which is capable of achieving higher photocatalytic activity.^19,20^ The components of the catalysts can be further confirmed by X-ray photoelectron spectroscopy (XPS, Fig. S8†).

For comparison, reference catalysts such as pure TiO_2_ hollow spheres (T-HSs) (Fig. S6a and b†) and TiO_2_/Pt/MnO_*x*_ hollow spheres (T/P/M-HSs) (Fig. S6c–f†) were synthesized by a similar method. PT-HSs were also used as reference catalysts. T/P/M-HSs were synthesized by impregnating T-HSs in solutions of H_2_PtCl_6_ and MnSO_4_ in sequence to form a structure in which the Pt particles and MnO_*x*_ distributed randomly on both the inner and outer surfaces.

**Fig. 5:**
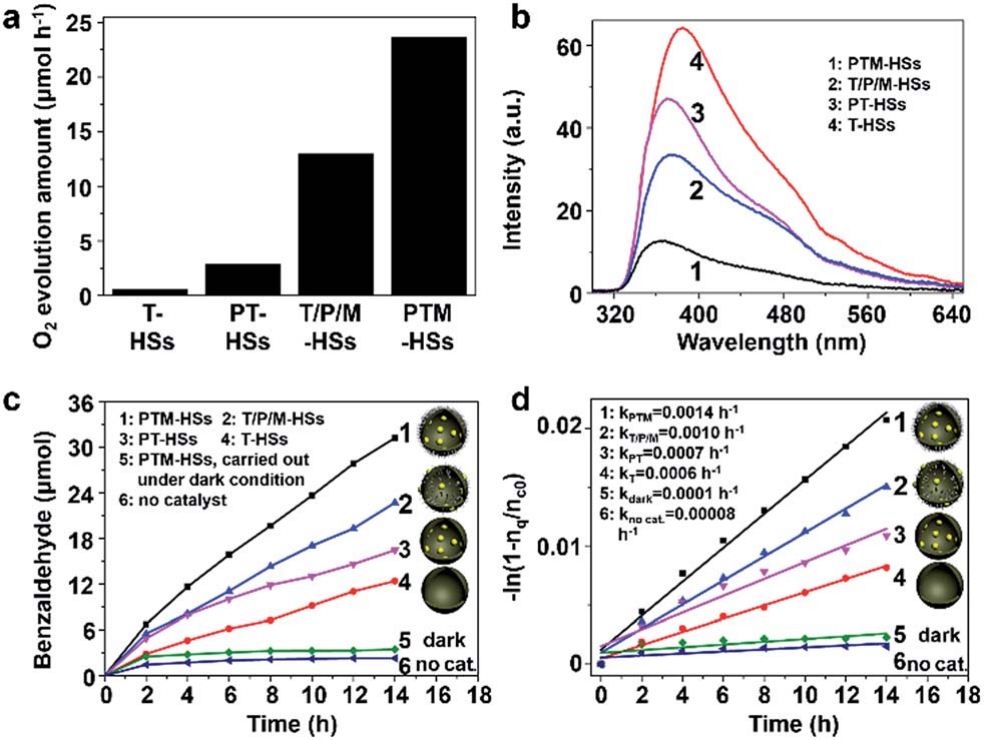
(a) The activity for photocatalytic water oxidation under visible light (*λ* > 420 nm). 0.03 g of catalyst was used. (b) The PL spectra (excited at *λ* = 235 nm) of diverse catalysts. (c) The activity for photocatalytic alcohol oxidation under UV light. (d) The corresponding kinetic rate plots. Traces 1–6 refer to PTM-HSs, T/P/M-HSs, PT-HSs, T-HSs, PTM-HSs under dark conditions, and no catalyst, respectively. Traces 1–4 and trace 6 are carried out under UV irradiation. 0.03 g of catalyst was used.

Catalysts containing Pt and TiO_2_ can exhibit activity in the photooxidation of alcohols (details are discussed in Fig. S12†) and water (Fig. 5a) under visible light, because of the surface plasmonic resonance effect (SPR) of Pt (Fig. S12e†).^17,21,22^ As shown in Fig. 5a, the highest water oxidation activity is achieved for the PTM-HSs. The deposited Pt on the inner surface can collect electrons to reduce IO_3_^–^ ions, and the MnO_*x*_ photo-deposited selectively on the outer surface can accumulate holes for water oxidation. For PT-HSs, although the amounts of loaded species and the topography of the catalysts are similar, the promotion effect of the cocatalysts is not as evident as that of PTM-HSs, indicating that the photocatalytic performance can be greatly enhanced when the reduction/oxidation cocatalysts are spatially separated. The activity of T-HSs and PT-HSs is even lower. These results can be explained by the improvement of the charge separation, which can be demonstrated by the photoluminescence (PL) spectra (Fig. 5b). The emission intensity of T-HSs is the strongest, resulting from the severe recombination of photogenerated charges. In contrast, the intensity of PT-HSs, T/P/M-HSs and PTM-HSs are reduced by about 25%, 45% and 80%, respectively, indicating an enhanced suppression of the recombination process.

To illustrate that the enhancement of activity is indeed caused by the improvement of charge separation, the oxidation of benzyl alcohol was performed under UV light (*λ* < 420 nm). UV light (1.46 mW cm^–2^) was adopted to ensure that light absorption remained the same (Fig. S7†) when different catalysts were used. Thus, the influence of light absorption can be eliminated. Benzyl alcohol was oxidized to benzaldehyde without any over-oxidized products such as benzoic acid (explained in Fig. S9†). Besides, the structure of the catalysts remained unchanged after the reaction, indicating excellent stability (Fig. S10†). As shown in Fig. 5c, negligible amounts of benzaldehyde were detected without the catalyst or in the dark condition (traces 5 and 6, Fig. 5c), indicating the importance of catalysts and light. The T-HSs display a moderate photocatalytic activity (trace 4, Fig. 5c, 12.39 μmol of product, 14 h), while the activity of PT-HSs is increased (trace 3, Fig. 5c, 22.72 μmol of product, 14 h). By loading Pt and MnO_*x*_ simultaneously (T/P/M-HSs), the activity is further enhanced (trace 2, Fig. 5c). The highest activity (31.22 μmol of product, 14 h) is achieved when PTM-HSs were used (trace 1, Fig. 5c). The corresponding study on kinetics (Fig. S14†) shown in Fig. 5d exhibits the same trend (*i.e.*, activity: PTM-HSs > T/P/M-HSs > PT HSs > T-HSs).

To further confirm the enhancement of separation efficiency (*η*_s_), the apparent quantum efficiency (AQE) of PTM-HSs and T/P/M-HSs at 254 nm were determined to be 63.14% and 45.94%, respectively, which is higher than that of conventional TiO_2_-based catalysts (often in the range 4.3–39%).^23^ Because of the similar structure of the two kinds of catalysts and the same loading amount of cocatalyst, the efficiency of adsorption (*η*_a_) and surface-reaction (*η*_c_) remain the same between the catalysts (Fig. S7†). Considering the overall efficiency is determined by *η*_a_, *η*_s_ and *η*_c_ simultaneously, the enhancement of AQE can be attributed to the enhancement of *η*_s_. Thus, we can quantitatively demonstrate that the spatial separation of cocatalysts indeed leads to an efficient charge separation for photocatalytic oxidation. Moreover, even under visible light (*λ* > 420 nm), the electrons (generated from catalysts containing Pt and TiO_2_) still flow from TiO_2_ to Pt (instead of the hot electrons injected into TiO_2_), which can be proved by a probe experiment (Fig. S12†).

**Fig. 6:**
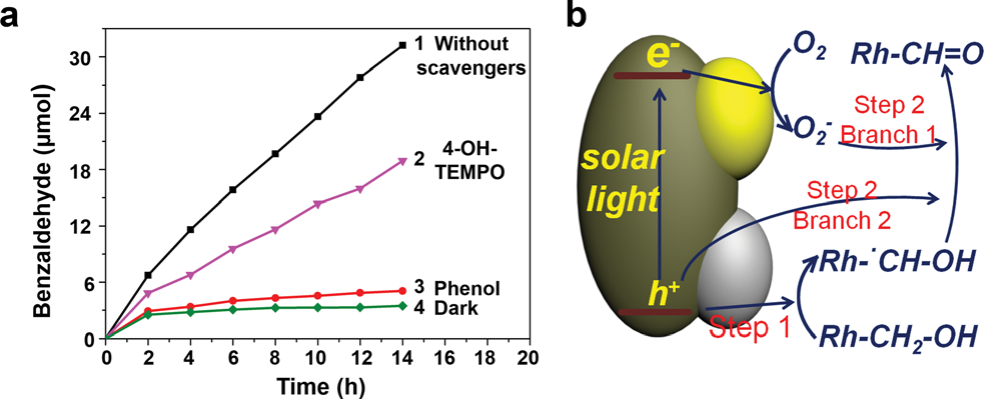
(a) Effects of diverse scavengers on the photooxidation of benzyl alcohol in the presence of PTM-HSs (0.03 g). Traces 1–3 were obtained under UV light, while trace 4 was under dark conditions. (b) The proposed mechanism of photocatalytic benzyl alcohol oxidation.

In order to investigate the mechanism of photocatalytic benzyl alcohol oxidation, particularly the main active species, phenol, and 4-hydroxy-2,2,6,6-tetramethylpiperidine 1-oxyl free radical (4-OH-TEMPO) were used as the scavengers of holes (h^+^) and superoxide radicals (O_2_^–^), respectively. As shown in Fig. 6a, when phenol (h^+^ scavenger) was added into the photocatalytic system, an extremely low oxidation activity was observed, indicating that h^+^ plays an indispensable role in the oxidation process. However, when 4-OH-TEMPO (O_2_^–^ scavenger) was added, the activity only partly decreased, indicating that O_2_^–^ also takes part in the reaction, while it is not indispensable. These observations suggest that two photocatalytic steps might be involved. The first step primarily depends on h^+^, while the second step is driven by h^+^ and O_2_^–^ simultaneously. An intermediate would be formed in step 1 and be oxidized in step 2, bridging the two steps.

To determine the intermediate species, a kinetic isotope effect (KIE) study was conducted for PTM-HSs with benzyl alcohol under aerobic conditions. The KIE value was 0.78, suggesting the breaking of a C–H bond instead of an O–H bond (see ESI†). Accordingly, the intermediate was determined to be Rh-˙CH–OH (Rh stands for the benzene ring). The speculated mechanism of photocatalytic benzyl alcohol oxidation is shown in Fig. 6b and eqn (1) to (5):
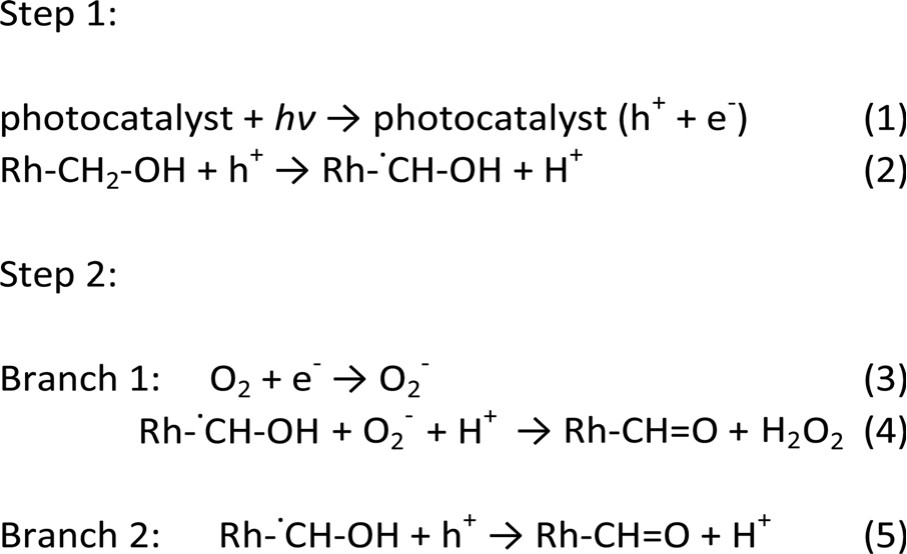


Upon the separation of photo-induced electrons and holes over the PTM-HSs, the holes react with benzyl alcohol molecules to form intermediate free radicals (Rh–˙CH–OH). Subsequently, some of the free radicals react with O_2_^–^, while others are further oxidized by holes to form the ultimate products (Rh–CH=O). Meanwhile, oxy species finally exist as H_2_O_2_ after a series of changes, which are discussed in detail in the ESI.† ^24^

## Conclusions

In summary, the PTM-HS catalyst with Pt and MnO_*x*_ loaded separately onto the inner and outer surfaces of TiO_2_ hollow spheres was successfully synthesized for the photooxidation of benzyl alcohol. Pt particles favor electron trapping, while MnO_*x*_ tends to collect holes. The spatial separation of Pt and MnO_*x*_ by the TiO_2_ shell greatly enhances the separation of electrons and holes. With this structure, different cocatalysts can be completely separated without intermixing. In addition, the large surface area of the TiO_2_ shell can provide plenary attachment sites for Pt and MnO_*x*_, accelerating the consumption of electrons, which is good for the oxidation of water and benzyl alcohol. Furthermore, considering the multiple improvements of light-absorption, charge-separation and surface catalytic effect, the structure of these PTM-HSs could provide inspiration for other photocatalytic systems such as water splitting and CO_2_ reduction.

## Experimental

### Materials

H_2_PtCl_6_·6H_2_O (99.9%) was purchased from Tianjin Kaiyingte chemical trade Co., Ltd. Tetraethyl orthosilicate (TEOS, 98%) was purchased from Tianjin Chemical Reagent No. 1 Plant. Titanium *tert*-butoxide (TBOT, ≥ 98.0%) and undecane (≥99.0%) were purchased from Sinopharm and Guangfu, respectively. Poly(4-vinylpyridine) (PVP), hydroxypropyl cellulose (HPC) and 4-hydroxy-2,2,6,6-tetramethylpiperidine 1-oxyl free radical (4-OH-TEMPO, >98%) were purchased from TCI. Toluene (≥99.5%), benzyl alcohol (≥98.0%) and benzaldehyde (≥98.5%) were purchased from Jiangtian. Phenol (99%) was purchased from J&K. Deionized water (18.25 MΩ cm) supplied by an UP Water Purification System was used in all experimental processes. All chemicals were obtained from commercial suppliers and used without further purification.

### Characterization

TEM was performed on a JEOL JEM 2100F electron microscope operating at 200 kV. The photoluminescence (PL) spectrum was performed on a Hitachi F-4600 fluorescence spectrophotometer. Crystalline structures were evaluated by XRD analysis using a Bruker D8 Focus operating at 40 kV and 40 mA equipped with nickel-filtered Cu Kα radiation (*λ* = 1.54056 Å). The BET surface area and pore structure of catalysts were measured using a Micromeritics Tristar 3000 analyzer by nitrogen adsorption at 77 K. The specific surface areas were calculated from the isotherms using the BET method. The pore distribution and the cumulative volumes of pores were obtained by the BJH method from the desorption branch of the adsorption isotherms. XPS was performed under ultrahigh vacuum (<10^–6^ Pa) on a Kratos XSAM 800 spectrometer with Mg Kα X-ray source (*E* = 1253.6 eV).

### Methods

#### Synthesis of Pt@TiO_2_ hollow spheres (PT-HSs)

TEOS (0.8 mL) was mixed with deionized water (5 mL), H_2_PtCl_6_·6H_2_O (0.2 g mL^–1^, 1 mL), ethanol (20 mL) and an aqueous solution of ammonia (0.4 mL). After stirring for 6 h at room temperature, the precipitated silica particles were separated by centrifugation and washed twice with ethanol, then re-dispersed in 5 mL of ethanol under sonication. Subsequently, the samples were vacuum dried at 80 °C overnight. Then they were calcined at 500 °C for 2 h under H_2_ atmosphere to form Pt particles anchored on the SiO_2_. Then such SiO_2_–Pt powders were dispersed in 5 mL ethanol under sonication overnight with shaking. Subsequently, the suspension was mixed with ethanol (20 mL), deionized water (0.1 mL) and HPC (0.1 g). After 30 min stirring, 5 mL of 2.84 M TBOT ethanol solution was injected into the mixture at a rate of 0.75 mL min^–1^. After injection, the temperature was increased to 85 °C with stirring under refluxing conditions for 100 min to give SiO_2_–Pt@TiO_2_ core–shell structures. The precipitate was isolated using centrifugation and washed twice with ethanol and water. The above SiO_2_–Pt@TiO_2_ composites were dispersed in 20 mL water into which 0.14 g of PVP was added. After 12 h, the precipitate was separated, re-dispersed in ethanol (20 mL), and then mixed with water (5 mL), TEOS (0.1 mL) and aqueous ammonia (0.4 mL) to form the SiO_2_ outermost protective layer. After stirring for 6 h, the resulting SiO_2_–Pt@TiO_2_@SiO_2_ composites were centrifuged, washed three times with ethanol and dried under 80 °C for 12 h. Subsequently, the above powders were calcined in air for 2 h followed by H_2_ for another 2 h at 500 °C. Then the calcined samples were dispersed in 20 mL water under sonication and heated to 70 °C. 1 mL of 1.67 M aqueous NaOH solution was added to the above suspension. After etching for 8 h, the PT-HSs were formed.

#### Synthesis of Pt@TiO_2_@MnO_*x*_ hollow spheres (PTM-HSs)

PT-HSs powders (0.05 g), MnSO_4_ solution (0.06 M, 5 mL) and NaIO_3_ solution (0.02 M, 5 mL) were mixed in 100 mL deionized water, and the suspension was then irradiated by a 300 W xenon lamp (*λ* < 420 nm, 1.46 mW cm^–2^) under continuous stirring. After 5 h photo-deposition, the precipitate was isolated using centrifugation, washed with deionized water more than 3 times, and finally dried at 60 °C overnight to give PTM-HSs structures.

#### Synthesis of TiO_2_ hollow spheres (T-HSs) and TiO_2_/Pt/MnO_*x*_ hollow spheres (T/P/M-HSs)

By repeating steps of the synthesis of Pt@TiO_2_ hollow spheres without adding H_2_PtCl_6_·6H_2_O, the T-HSs were synthesized. To synthesize the T/P/M-HSs, a SiO_2_–TiO_2_ core–shell structure was synthesized at first. Then the samples were mixed with H_2_PtCl_6_ solution (0.01 g mL^–1^, 20 mL). After 12 h stirring, the suspension was vacuum dried at 80 °C overnight. Then SiO_2_ was re-coated as the outermost layer. Then the samples were calcined in air and H_2_ and etched in NaOH using methods mentioned in the step of the synthesis of Pt@TiO_2_ hollow spheres. Finally, MnO_*x*_ was loaded by the photo-deposition method mentioned above.

#### Photocatalytic oxidation of benzyl alcohol to benzaldehyde

Typically, 0.03 g of photocatalysts (PTM-HSs, T/P/M-HSs, PT-HSs or T-HSs) were added in a home-made reactor sealed with rubber stoppers. Then 5 mL of toluene (solvent), 50 μL of benzyl alcohol (reactant) and 40 μL of undecane (internal standard) were injected into the reactor. O_2_ was bubbled through the mixture at 20 mL min^–1^. The reactor was irradiated under magnetic stirring using a 300 W xenon lamp (PerfectLight Co.) to provide ultraviolet light (*λ* < 420 nm) with an irradiation area of 6.25 cm^2^. The irradiation intensity was 1.46 mW cm^–2^. Cooling water was used to eliminate the thermal effect in the reaction. The products were analyzed by a gas chromatograph system (GC 2060, Ramiin) with a flame ionization detector (FID). A standard solution (a mixture of 5 mL of toluene, 50 μL of benzyl alcohol, 40 μL of benzaldehyde and 40 μL of undecane) was used to calibrate the GC. The calibration factor (1.7) was obtained using the standard solution. Subsequently, the accurate amount of every sample was calculated using the area of every peak and the calibration factor. The correlation of peaks and products is determined by the retention time obtained from the prior analysis of pure chemicals, and further confirmed by the GC-MS analysis (Fig. S11†). To investigate the mechanism, 0.06 g of phenol and 0.06 g of 4-OH-TEMPO were added to the mixture containing 0.03 g of PTM-HSs, 5 mL of toluene (solvent), 50 μL of benzyl alcohol (reactant) and 40 μL of undecane (internal standard). Subsequent reactions and detections were similar to those above.

#### Photocatalytic oxidation of water to O_2_

The photocatalytic O_2_ evolution reactions were carried out in a closed gas circulation and evacuation system using a 300 W xenon lamp (PerfectLight Co.) and an optical cutoff filter (PerfectLight Co. *λ* > 420 nm). Normally, 0.03 g of photocatalyst was dispersed in 50 mL of 0.02 M NaIO_3_ aqueous solution in a glass reaction cell. Before irradiation, the reaction system was thoroughly degassed by evacuation in order to drive off the air inside. The amount of evolved O_2_ was determined by an online GC (TCD, Ar carrier). The rate of O_2_ evolution in the initial 3 h was recorded for comparison.

#### Apparent quantum efficiency (AQE)

The AQE was calculated with the following equation: AQE (%) = *N*_e_/*N*_p_, where *N*_e_ and *N*_p_ are the amounts of electrons (taking part in the reaction) and incident photons, respectively. The amount of electrons is two times the amount of benzaldehyde, for every benzaldehyde molecule consumes two electrons during the reaction. The amount of benzaldehyde per unit mass and unit time was measured by a GC system after reaction under irradiation with the wavelength at *λ* = 254 nm given by xenon lamp and a glass filter. The light intensity was fixed at 0.15 mW cm^–2^ (measured by an irradiatometer, UV-A, Photoelectric Instrument Factory of Beijing Normal University), and the amount of incident photons per unit time was calculated by the intensity and the energy of a single photon at *λ* = 254 nm.

#### Turnover number (TON)

TON = (the number of transformed substrate molecules)/(the number of active sites). The number of transformed substrate molecules can be obtained by GC analysis, which has been discussed above (page 4 of ESI†). When calculating the denominator, it should be noticed that only cocatalysts (Pt and MnO_*x*_) loaded onto TiO_2_ act as active sites, which could be illustrated by the probe experiment (Fig. S12†). The estimated TON is 4.6.
