# Supplementary material for: Spatial separation of oxidation and reduction co-catalysts for efficient charge separation: Pt@TiO2@MnO x hollow spheres for photocatalytic reactions
Source: Chem Sci. 2015 Nov 26;7(2):890–5. doi: 10.1039/c5sc04163e (PMC5530359; doi:10.1039/c5sc04163e)
Supplement: Supplementary file 1 [file SC-007-C5SC04163E-s001.pdf]

Electronic Supplementary Material (ESI) for Chemical Science.
This journal is © The Royal Society of Chemistry 2015

Supporting information

Spatial Separation of Oxidation and Reduction Co-catalysts for Efficient Charge Separation: Pt@TiO_2@MnO_x Hollow Spheres for Photocatalytic Reactions

Ang Li, Tuo Wang, Xiaoxia Chang, Weiting Cai, Peng Zhang, Jijie Zhang and Jinlong Gong*

Key Laboratory for Green Chemical Technology of Ministry of Education, School of Chemical Engineering and Technology, Tianjin University; Collaborative Innovation Center of Chemical Science and Engineering, Tianjin 300072, China

*Email: jlgong@tju.edu.cn

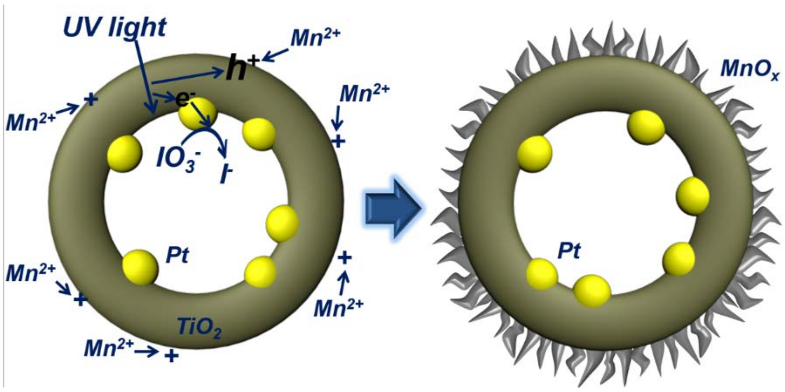

Supplementary Figure S1. The formation process of PTM-HSs. $NaIO_3$ and $MnSO_4$ were used as precursors during the deposition. Under irradiation, electrons were generated from TiO_2, and then trapped by Pt particles loaded on the inner surface. The electrons would be consumed by IO_3^-, leaving holes on the outer surface of TiO_2 shell. Mn^{2+} would react with holes to form the outermost spine-like MnO_x layer. The reactions can be summarized in equations (S1) and (S2):[1]

$$Mn^{2+} + xH_2O + (2x\text{-}2)\ h^+ \rightarrow MnO_x + 2xH^+ \qquad (S1)$$

$$IO_3^- + 6e^- \rightarrow I^- + 3/2O_2 \qquad (S2)$$

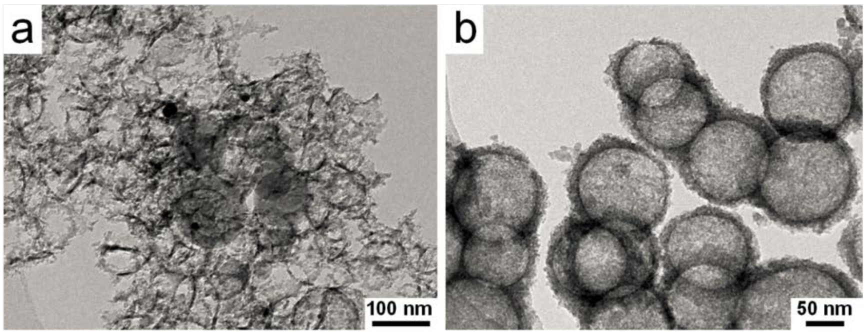

Supplementary Figure S2. TEM images of (a) crashed PT-HSs without the SiO_2 outermost protective layers and (b) normal PT-HSs with the outermost protective layers during synthesis.

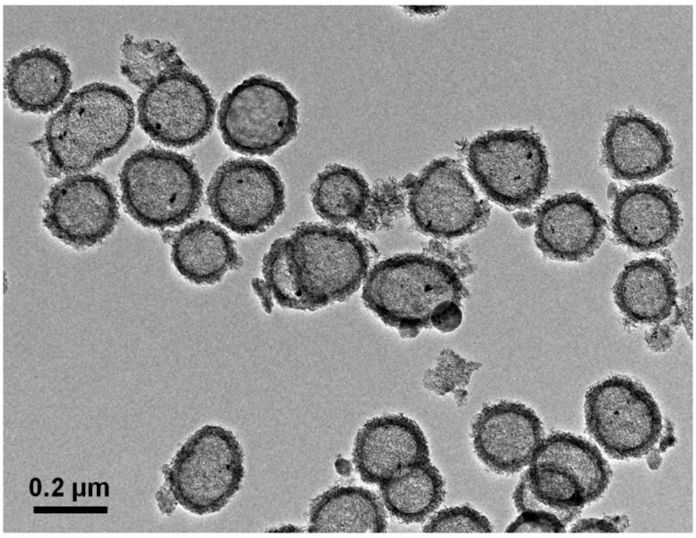

Supplementary Figure S3. The TEM image of PTM-HSs containing a larger area.

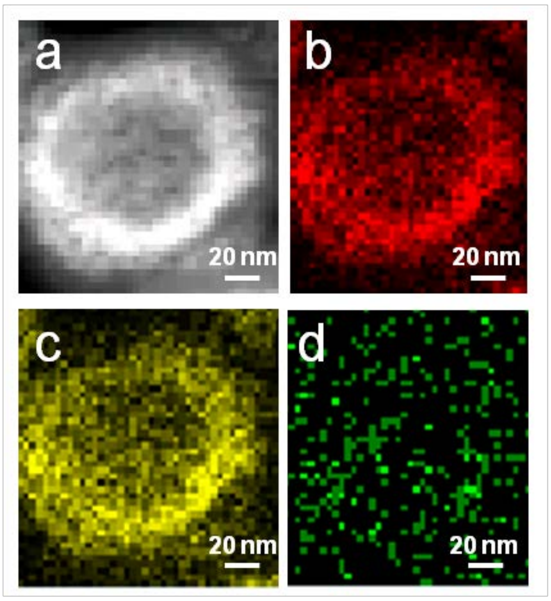

Supplementary Figure S4. The EDS area scan of PT-HSs. (a) TEM image of PT-HSs. (b, c, e) refer to the signal of Ti, O and Pt, respectively.

The distribution of Ti and O can prove that the shell consists of Ti and O, while the distribution of Pt cannot be exhibited clearly. Because of the low loading amount, the signal of Pt cannot be distinguished from the background noise.

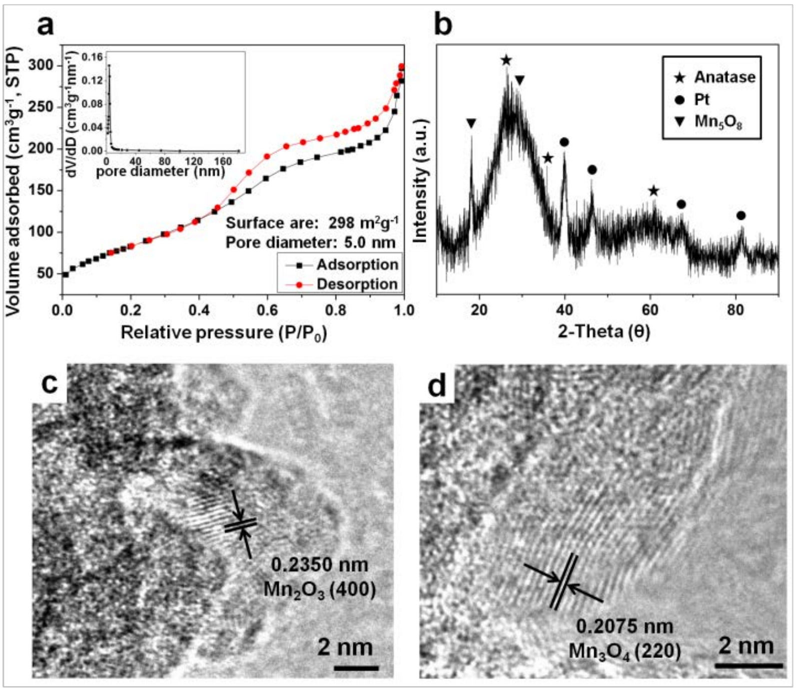

Supplementary Figure S5. (a) Nitrogen adsorption isotherms and pore size distribution (insert) of PTM-HSs. The pattern presents a type IV isotherm with a well-defined hysteresis loop, which indicates the well-developed mesoporous characteristic. In the inset, the sharp peak at about 4.0 nm suggests a relatively narrow pore size distribution. The average pore size is determined to be 5.0 nm. (b) XRD pattern of PTM-HSs. There are three series of peaks, which can be attributed to anatase TiO_2, Pt particles and Mn_5O_8, respectively. Meanwhile, some weak peaks in XRD pattern can be attributed to other types of oxides of manganese such as Mn_2O_3 and Mn_3O_4. (c, d) HRTEM images of the outermost spine-like layer. The planar spaces of 0.2350 nm and 0.2075 nm well match the (400) planes of Mn_2O_3 and (220) planes of Mn_3O_4.

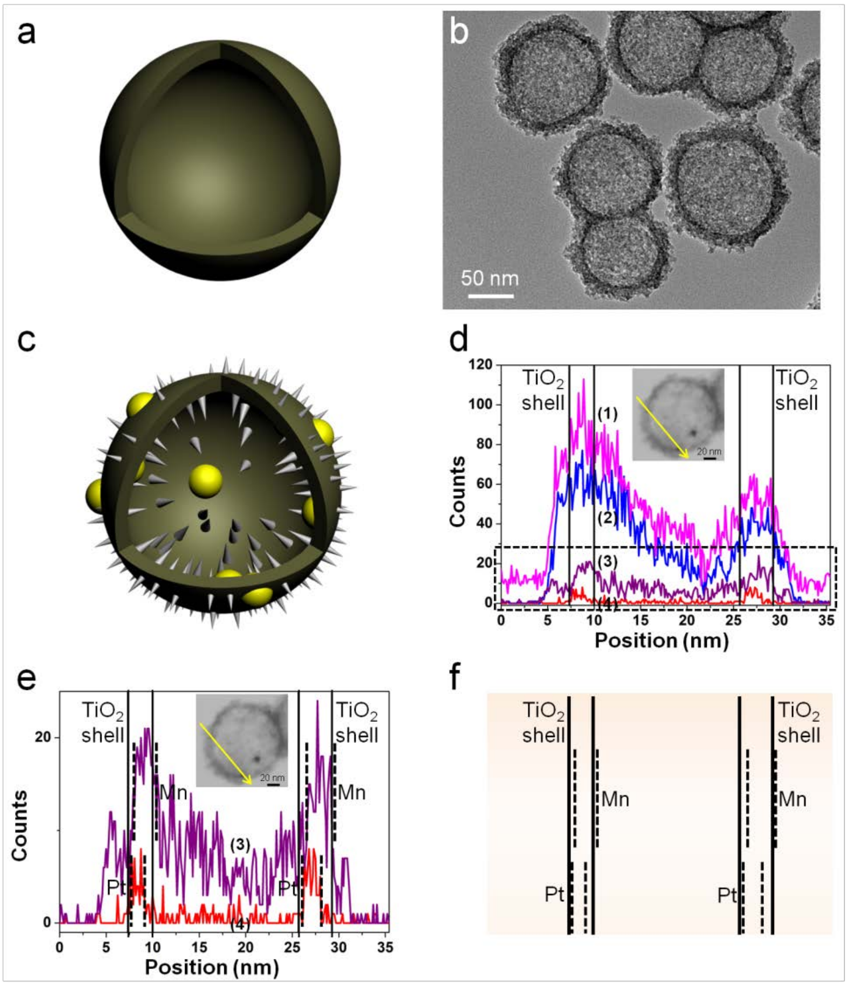

Supplementary Figure S6. The demonstration of T-HSs and T/P/M-HSs structures. (a) The schematic illustration of T-HSs. (b) TEM image of T-HSs. (c) The schematic illustration of T/P/M-HSs. (d) EDS line scan of T/P/M-HSs. The part in doted pane is magnified in image e. (e) The magnified patterns of the EDS line scan. (f) The relative position of Mn, Pt and TiO_2 shell. Inset in image d and e: the path of EDS line scan. Curve (1), (2), (3) and (4) in image d and e refer to the signal of Ti, O, Mn and Pt, respectively.

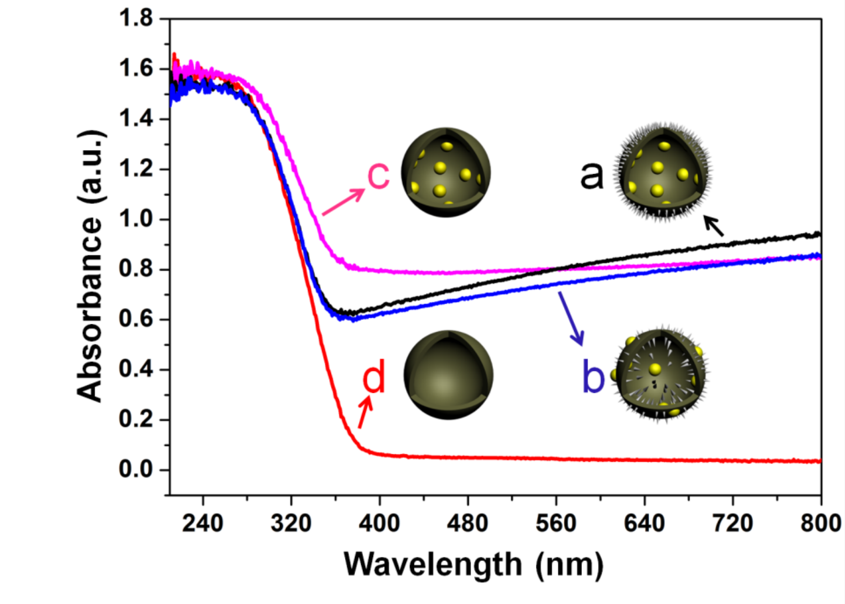

Supplementary Figure S7. UV-Vis absorption spectra of diverse catalysts. (a) PTM-HSs. (b) T/P/M-HSs. (c) PT-HSs. (d) T-HSs.

All the catalysts present strong absorption during the UV region ($\lambda < 420$ nm). It can be concluded that the efficiency of absorption (η_a) of PTM-HSs and PTM-HSs remains the same.

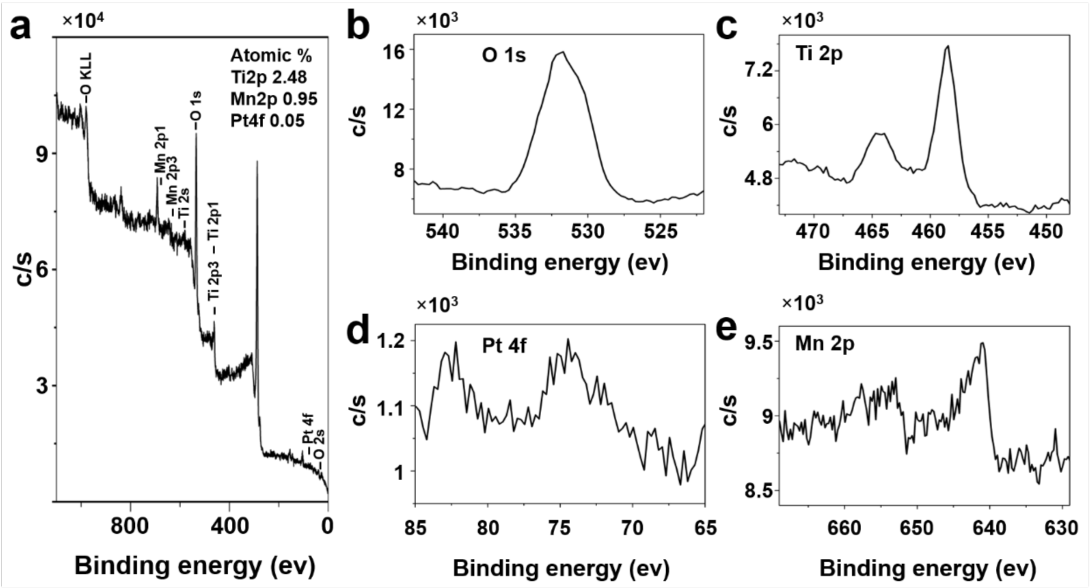

Supplementary Figure S8. XPS of PTM-HSs. (a) the overall pattern and atomic percent. (b) O 1s. (c) Ti 2p. (d) Pt 4f. (e) Mn 2p.

The atomic ratio of Pt:Ti:Mn is 1:50:19. According to the binding energies of Pt 4f and Mn 2p, the deposited manganese species can be ascribed to MnO_x, where x is between 1.0 and 2.0.[1]

.

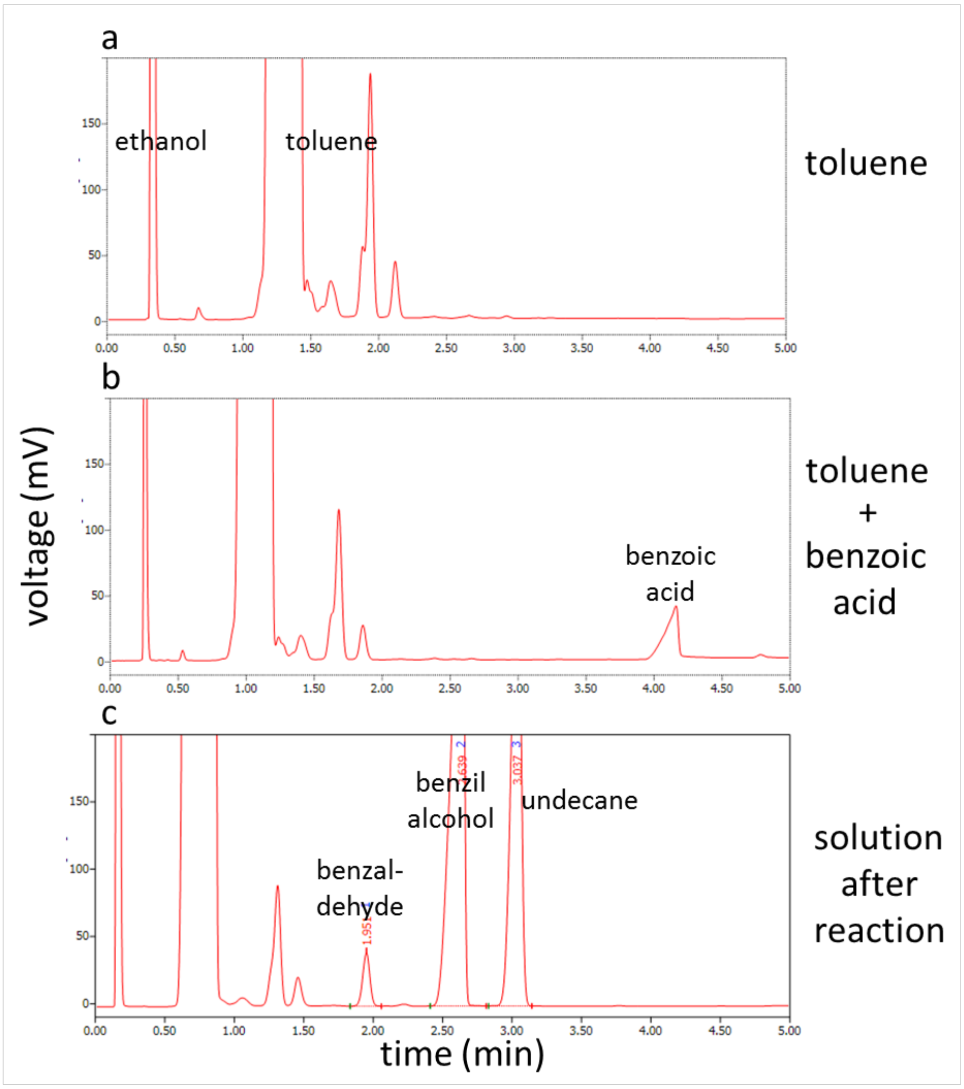

Supplementary Figure S9. Chromatographic detection results of (a) toluene; (b) mixture of toluene and benzoic acid; (c) solution after reaction with PTM-HSs (14 h).

Ethanol was used to wash the injection needle. The comparison of a and b indicates that the retention time of benzoic acid is about 4 min. c does not show any peaks at 4 min, indicating that there are no benzoic acids generated during the reaction. Actually, because benzyl alcohol is easier to be adsorbed on catalysts than benzaldehyde, benzaldehyde may desorb from catalysts after formation, inhibiting the further oxidation.

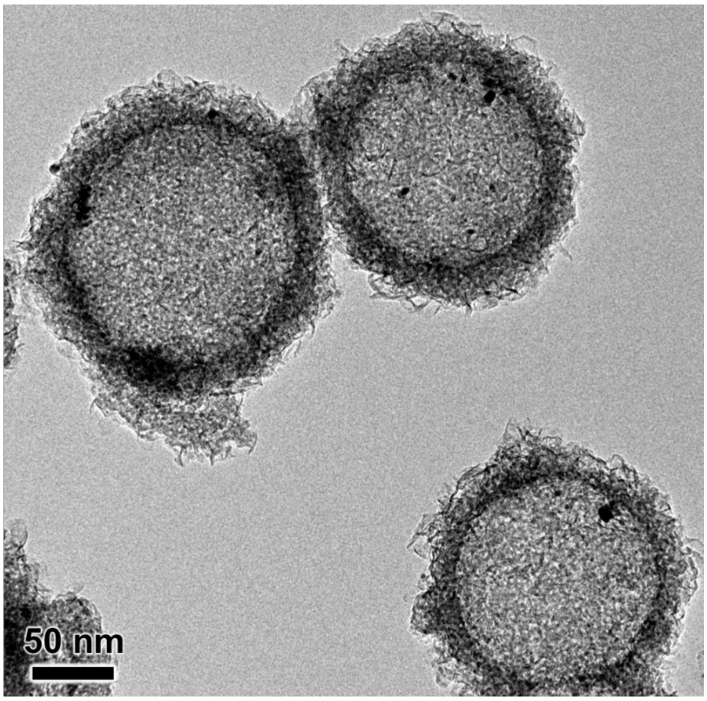

Supplementary Figure S10. TEM image of PTM-HSs after the oxidation reaction of benzyl alcohol (30 mg catalysts, 14h, UV light, the irradiation intensity is 1.46 mW cm^{-2}).

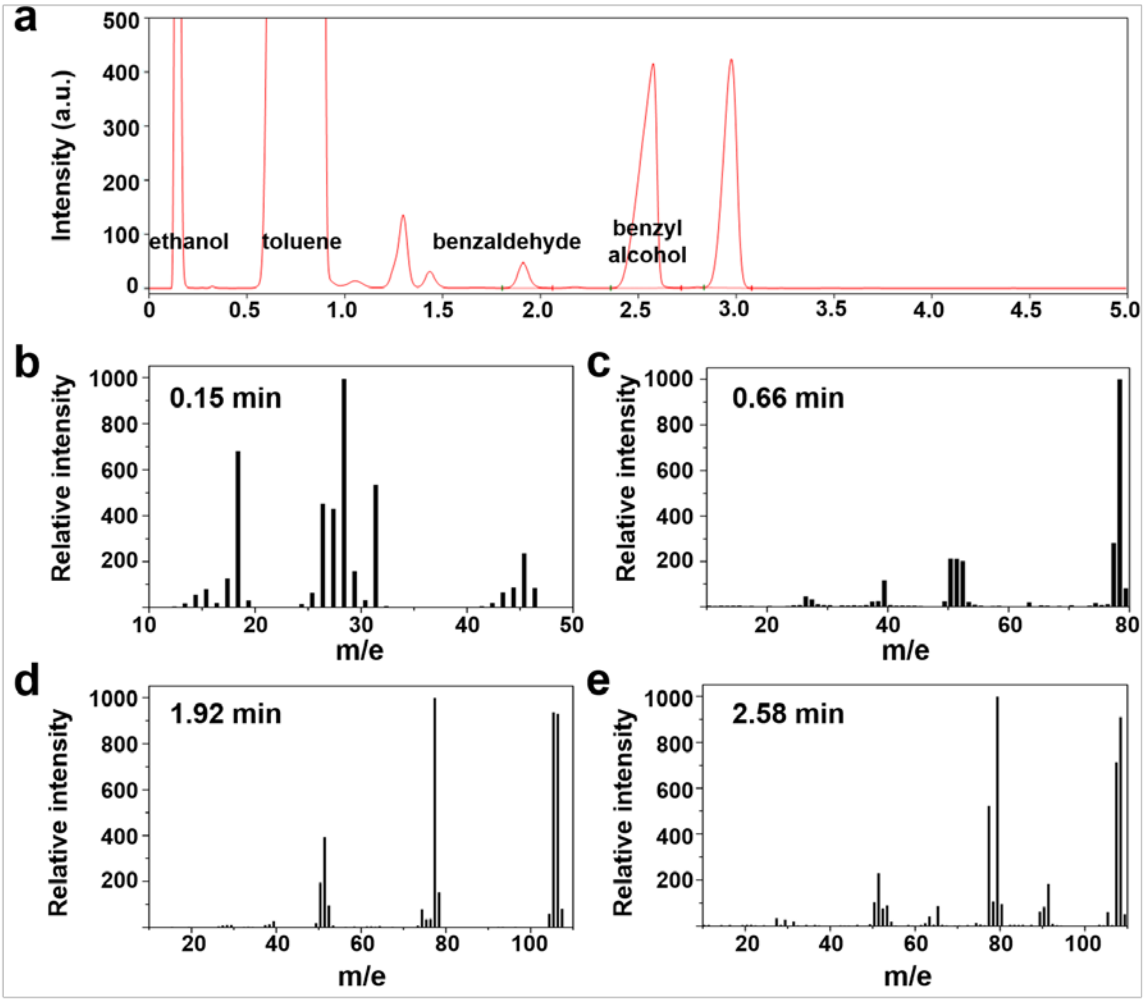

Supplementary Figure S11. The GC-MS results. (a) The result of products analysis by GC-FID. (b) The GC-MS pattern of 0.15 min after injection. (c) 0.66 min. (d) 1.92 min. (e) 2.58 min.

The results of GC-MS further confirm that the peaks at 0.15 min, 0.66 min, 1.92 min and 2.98 min stand for ethanol, toluene, benzaldehyde and benzyl alcohol, respectively, ensuring the validity of the product analysis method, which has been discussed above (page 4 of SI, *Photocatalytic oxidation of benzyl alcohol to benzaldehyde*).

The activity under visible light

Different from pure TiO_2 nanospheres, catalysts containing Pt and TiO_2 exhibit visible-light activity because of the surface plasmonic resonance effect (SPR) of metal nanoparticles. However, under visible light, the migration direction of electrons remain unclear. Some believe that electrons still transfer from semiconductor to metal particles in despite of SPR,[2-4] while others hold the opposite opinions.[5] Thus, a probe experiment was conducted to investigate the flow direction of electrons.[6]

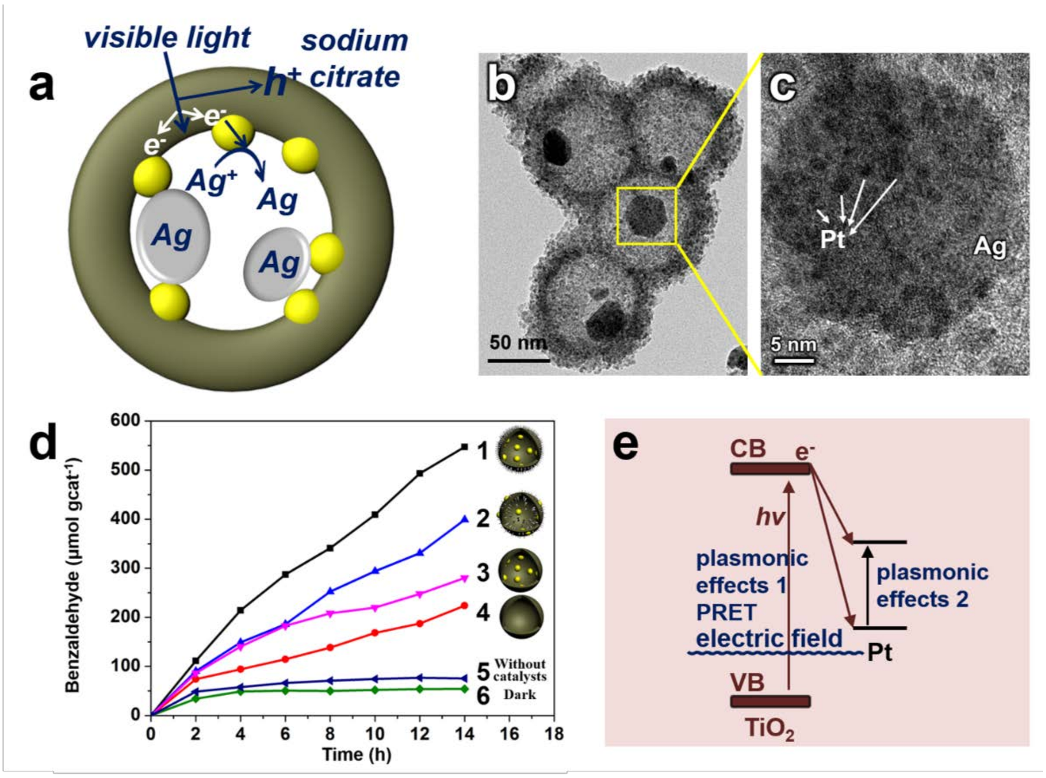

Supplementary Figure S12. (a) Scheme of the probe experiment. (b, c) TEM and HRTEM images of the PT-HSs after the probe experiment. (d) The activity of photocatalytic oxidation under visible light ($\lambda > 420$ nm) with diverse catalysts. Trace 1: PTM-HSs. Trace 2: T/P/M-HSs. Trace 3: PT-HSs. Trace 4: T-HSs. Trace 5: without catalysts. Trace 6: PTM-HSs, carried out without light. Traces 1-5 are carried out under visible irradiation. (e) The process of plasmon-enhanced photooxidation under visible light. PRET refers to the plasmon resonance energy transfer, which is caused by a plasmonic electric field.

Ag^+ (from $AgNO_3$) ion was used as a probe ion and sodium citrate as a positive charge scavenger. Under visible light ($\lambda > 420$ nm), Ag particles would be formed where electrons are accumulated, indicating the migration direction of electrons (Fig. S12a). Holes would be consumed by sodium citrate. TEM image (Fig. S12b) shows that large Ag particles are only formed on the inner side of TiO_2 shells, where Pt particles are located. Pt particles are only located on the inner surface of TiO_2 shell instead of the outer side, indicating that Ag particles only grown on Pt particles. Besides, from the HRTEM (Fig. S12c) we can see that Ag particles are indeed formed near Pt particles. The results suggest that electrons still immigrate form TiO_2 to Pt particles even under visible light, which is similar with the migration direction of electrons under UV light. Fig. S12d shows that under visible light PTM-HSs exhibit the highest activity, followed by T/P/M-HSs, PT-HSs and T-HSs, which are also similar with the activities under UV light. Such results suggest the same enhancement of η_s by the spatial separation of cocatalysts even under visible light.

The process of such plasmon-enhanced reaction under visible light can be described by Fig. S12e. The process of collective plasmon resonance mainly follow two pathways: the plasmon resonance energy transfer (PRET) and the production of hot electrons.[7] PRET is caused by a plasmonic electric field, which transfers energy from plasmonic metal to a nearby semiconductor, improving the separation of electrons and holes.[7] As for the hot electrons, they are produced by the rise of fermi level of metals. If the fermi level is higher than the conduction band (CB) of TiO_2, electrons will be injected into the semiconductor. However, in this study, electrons transfer freely from TiO_2 to Pt (which has been proved by the above probe experiment), suggesting that fermi level of Pt is still lower than the CB of TiO_2.[8]

Details of the probe experiment: Typically, 0.03 g PT-HSs were mixed with 60 mL $AgNO_3$ solution (0.026 mol L^{-1}). 1.2 g sodium citrate was added into this suspension. After 0.5 h stirring, the mixture was irradiated by a 300 W Xe lamp ($\lambda >$ 420 nm) for 5 h with 80 mW cm^{-2}. The suspension was centrifuged and washed by deionized water for three times.

The change of oxy species

Oxy species finally exist as H_2O_2 after a series of change as shown in equations S3 to S5.[9] e^- is provided by photocatalysts and H^+ is provided by Rh-CH_2-OH.

$$O_2^{-} + H^{+} \rightarrow HOO^{\cdot} \quad \text{(S3)}$$

$$HOO^{\cdot} + e^{-} \rightarrow HO_2^{-} \quad \text{(S4)}$$

$$HOO^{-} + H^{+} \rightarrow H_2O_2 \quad \text{(S5)}$$

The study on kinetics and KIE to determine the form of intermediate

According to the proposed mechanism, an intermediate would be formed after step 1 and be oxidized in step 2. Considering the structure of benzyl alcohol and benzaldehyde, it is easy to infer that in step 1, C-H or O-H bond of $-CH_2$-OH will break to form the intermediate free radical. This kind of free radical is not stable and it will react with oxidants very rapidly to form C=O double bond in step 2, suggesting that step 1 is the rate-determining step.[10] However, because the kind of broken bond in step 1 remains unknown, the intermediate free radical has two possible forms: Rh-$^{\cdot}$CH-OH (Fig. S13a) and Rh-CH_2-O$^{\cdot}$ (Fig. S13b). To determine the specific form, experiments based on kinetic isotope effect were conducted.

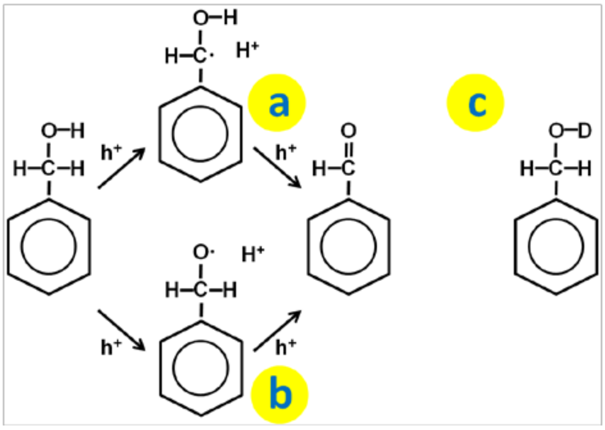

Supplementary Figure S13. Possible forms of the intermediate and the structure of as-prepared deuterium-benzyl alcohol. (a) Rh-$^{\cdot}$CH-OH. (b) Rh-CH_2-O$^{\cdot}$. (c) Rh-CH_2-OD.

The kinetic isotope effect (KIE) is the change in the rate of a chemical reaction when one of the atoms in the reactants is substituted with one of its isotopes. To describe the change quantitatively, KIE is expressed as the ratio of rate constants for the reactions involving the light (k_{light}) and the heavy (k_{heavy}) isotopically substituted reactants: KIE= k_{light}/k_{heavy}. Changing a hydrogen atom (H) to its isotope deuterium (D) is known as the hydrogen isotope effect. In general, the hydrogen kinetic isotope effect can be classified into primary hydrogen kinetic isotope effect (PHKIE) and secondary hydrogen kinetic isotope effect (SHKIE). The PHKIE refers to cases in which a bond to the isotopically labeled hydrogen is formed or broken, while the SHKIE arises in cases where the isotopic substitution is remote from the bond being broken. The remote

atom, nonetheless, influences the internal vibrations of the system and then will affect the rates of chemical reactions. Typically, the KIE of PHKIE is located between 2 and 7, while SHKIE between 0.7 and 1.5.[10]

Herein we have synthesized the deuterium-benzyl alcohol (Rh-CH_2-OD, Fig. S13c) by replacing the hydrogen of oxhydryl with deuterium. The Rh-CH_2-OD was oxidized under the same condition as the reaction of Rh-CH_2-OH over PTM-HSs. Generally, the oxidation of benzyl alcohol can be regarded as a first-order reaction, which can also be proved by Fig. S14. By a series of conversions, the kinetic relationship can be expressed by the initial amount of benzyl alcohol (n_{c0}), the amount of benzaldehyde (n_q) and reaction time (t) as shown in Equation S6:

$$-\ln(1-\frac{n_q}{n_{c0}})=kt \qquad \text{(S6)}$$

Fig. S14 is constructed according to Equation S6, and the slopes of curves stand for the rate constants. The KIE is determined to be 0.78, suggesting that the KIE of such reaction belongs to SHKIE. The result indicates that the O-H bond remains unbroken in the first step, so the intermediate can be identified as Rh-$^{\cdot}$CH-OH.

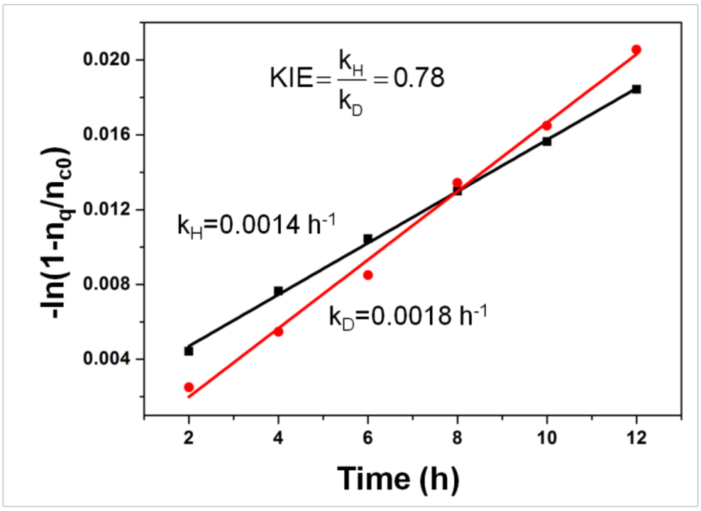

Supplementary Figure S14. k_H and k_D stand for the rate constants of the reactions with benzyl alcohol and deuterium-benzyl alcohol, respectively.

Methods: 0.5 g sodium (99.5%, J&K) reacted with 7.5 mL Deuterium oxide (99.8%atomD, TCI) to generate NaOD. Then NaOD was mixed with 5 mL benzyl chloride, then the temperature was increased to 95°C with stirring under refluxing conditions for 72 h to give Rh-CH_2-OD. Then the Rh-CH_2-OD was oxidized under the same condition as the reaction of Rh-CH_2-OH over PTM-HSs. The products were analyzed by a gas chromatograph system (GC 2060, Ramiin) with a flame ionization detector (FID).

References

1 R. Li, F. Zhang, D. Wang, J. Yang, M. Li, J. Zhu, X. Zhou, H. Han and C. Li, *Nat. Commun.*, 2013, **4**, 1432-1438.
2 S. Obregón and G. Colón, *Appl. Catal. B: Environ.*, 2014, **144**, 775-782.
3 J. Fang, L. Yin, S. Cao, Y. Liao and C. Xue, *Beilstein J. Nanotechnol.*, 2014, **5**, 360-364.
4 J. Hou, H. Cheng, O. Takeda and H. Zhu, *Angew. Chem. Int. Ed. Engl.*, 2015, **54**, 8480-8484.
5 Y. Shiraishi, D. Tsukamoto, Y. Sugano, A. Shiro, S. Ichikawa, S. Tanaka and T. Hirai, *ACS Catal.*, 2012, **2**, 1984-1992.
6 A. Li, P. Zhang, X. Chang, W. Cai, T. Wang and J. Gong, *Small*, 2015, **11**, 1892-1899.
7 S. C. Warren and E. Thimsen, *Energ. Environ. Sci.*, 2012, **5**, 5133-5146.
8 W. N. Wang, W. J. An, B. Ramalingam, S. Mukherjee, D. M. Niedzwiedzki, S. Gangopadhyay and P. Biswas, *J. Am. Chem. Soc.*, 2012, **134**, 11276-11281.
9 J. C. Colmenares and R. Luque, *Chem. Soc. Rev.*, 2014, **43**, 765-778.
10 F. A. Carey, R. J. Sundberg, *Advanced Organic Chemistry, Part A: Structure and Mechanisms*, Springer, New York, **1993**.
